# Saturated fat, the estimated absolute risk and certainty of risk for mortality and major cancer and cardiometabolic outcomes: an overview of systematic reviews

**DOI:** 10.1186/s13643-023-02312-3

**Published:** 2023-09-30

**Authors:** Jhalok Ronjan Talukdar, Jeremy P. Steen, Joshua Z. Goldenberg, Qian Zhang, Robin W. M. Vernooij, Long Ge, Dena Zeraatkar, Małgorzata M. Bała, Geoff D. C. Ball, Lehana Thabane, Bradley C. Johnston

**Affiliations:** 1 Department of Health Research Methods, Evidence, and Impact, McMaster University, Hamilton, ON Canada; 2 Faculty of Health Sciences, McMaster University, Hamilton, ON Canada; 3 Department of Nutrition, College of Agriculture and Life Sciences, Texas A&M University, College Station, TX USA; 4 Helfgott Research Institute, National University of Natural Medicine, Portland, OR USA; 5 School of Nursing, Faculty of Health Sciences, University of Ottawa, Ottawa, ON Canada; 6 Department of Nephrology and Hypertension, University Medical Center Utrecht, Utrecht, The Netherlands; 7 Julius Center for Health Sciences and Primary Care, University Medical Center Utrecht, Utrecht University, Utrecht, The Netherlands; 8 Evidence Based Social Science Research Centre, School of Public Health, Lanzhou University, Lanzhou, China; 9 Department of Biomedical Informatics, Harvard Medical School, Boston, MA USA; 10 Department of Hygiene and Dietetics, Chair of Epidemiology and Preventive Medicine, Jagiellonian University Medical College, Krakow, Poland; 11 Department of Pediatrics, Faculty of Medicine & Dentistry, College of Health Sciences, University of Alberta, Edmonton, AB Canada; 12 Faculty of Health Sciences, University of Johannesburg, Johannesburg, South Africa; 13 Biostatistics Unit, St Joseph’s Healthcare-Hamilton, Hamilton, ON Canada; 14 Department of Nutrition, College of Agriculture and Life Sciences, Texas A&M University, College Station, TX USA; 15 Department of Epidemiology and Biostatistics, School of Public Health, Texas A&M University, College Station, TX USA

**Keywords:** Nutrition, Saturated fatty acids, Cardiovascular disease, Cancer, Systematic review

## Abstract

**Objective:**

To assess the impact of reducing saturated fat or fatty foods, or replacing saturated fat with unsaturated fat, carbohydrate or protein, on the risk of mortality and major cancer and cardiometabolic outcomes in adults.

**Methods:**

We searched MEDLINE, EMBASE, CINAHL, and references of included studies for systematic reviews and meta-analyses (SRMAs) of randomized controlled trials (RCTs) and observational studies in adults published in the past 10 years. Eligible reviews investigated reducing saturated fat or fatty foods or replacing saturated fat with unsaturated fat, carbohydrate or protein, on the risk of cancer and cardiometabolic outcomes and assessed the certainty of evidence for each outcome using, for example, the GRADE (Grading of Recommendations, Assessment, Development, and Evaluations) approach. We assessed the quality of SRMAs using a modified version of AMSTAR-2. Results were summarized as absolute estimates of effect together with the certainty of effects using a narrative synthesis approach.

**Results:**

We included 17 SRMAs (13 reviews of observational studies with follow-up 1 to 34 years; 4 reviews of RCTs with follow-up 1 to 17 years). The quality of two-thirds of the SRMAs was critically low to moderate; the main limitations included deficient reporting of study selection, absolute effect estimates, sources of funding, and a priori subgroups to explore heterogeneity. Our included reviews reported > 100 estimates of effect across 11 critically important cancer and cardiometabolic outcomes. High quality SRMAs consistently and predominantly reported low to very low certainty evidence that reducing or replacing saturated fat was associated with a very small risk reduction in cancer and cardiometabolic endpoints. The risk reductions where approximately divided, some being statistically significant and some being not statistically significant. However, based on 2 moderate to high quality reviews, we found moderate certainty evidence for a small but important effect that was statistically significant for two outcomes (total mortality events [20 fewer events per 1000 followed] and combined cardiovascular events [16 fewer per 1000 followed]). Conversely, 4 moderate to high quality reviews showed very small effects on total mortality, with 3 of these reviews showing non-statistically significant mortality effects.

**Conclusion:**

Systematic reviews investigating the impact of SFA on mortality and major cancer and cardiometabolic outcomes almost universally suggest very small absolute changes in risk, and the data is based primarily on low and very low certainty evidence.

**Systematic review registration:**

PROSPERO CRD42020172141

**Supplementary Information:**

The online version contains supplementary material available at 10.1186/s13643-023-02312-3.

## Introduction

Non-communicable diseases, including cardiovascular disease (CVD), cancer, and diabetes are responsible for 4 out of 5 deaths worldwide [1], with unhealthy dietary habits often listed as a leading risk factor for premature death [2, 3]. Unhealthy dietary habits are often described as a high intake of fat in general and saturated fatty acids (SFA) in particular [4, 5]. Higher intake of SFA can promote oxidative stress and inflammation, which can increase the risk for some cancers, as well as an increase in low-density lipoprotein (LDL) cholesterol, a biomarker associated with CVD. Reducing dietary fat intake, often targeted specifically to SFA, has been the orthodox position in the nutrition community since the 1950s following the Seven Country Study and the ‘diet-heart hypothesis’ [6].

However, dietary guidelines that recommend reducing SFA have been subject to increasing scrutiny as further evidence has accumulated [7]. Questions have been raised regarding the quality of evidence to support population-level guidelines advocating for SFA reduction [8]. Specifically, while some evidence supports the link between the reduction of SFA and the subsequent reduction in surrogate outcomes such as LDL cholesterol, including replacing SFA with polyunsaturated fatty acids (PUFAs) [9], direct evidence between dietary SFA change and the risk of patient and public important health outcomes such as cancer and cardiovascular mortality is sparse and subject to methodologic flaws and questionable inferences [10, 11], with some nutrition researchers now questioning the orthodox view [12]. The strength of the evidence supporting the orthodox position depends on a number of nutrition-specific and methodologic considerations, including 1) what SFA is replaced with in the diet (e.g., PUFA, monounsaturated fatty acids (MUFA), carbohydrates, protein); 2) focusing on a single nutrient versus foods that include SFA, PUFA, MUFA, and other nutrients [12]; 3) the methodological quality of systematic reviews of the evidence; 4) the overall certainty of the evidence for patient and public important outcomes and; 5) whether both relative and absolute estimates of effects are calculated and presented. We sought to address these considerations by conducting an overview of systematic reviews to assess the impact of reducing saturated fat or fatty foods, or replacing saturated fat with unsaturated fat, carbohydrate or protein, on the risk of cancer and cardiometabolic outcomes in adults with varying cardiometabolic risk factors.

## Material and methods

Our overview of SRMAs followed an a priori protocol (CRD42020172141) based on guidance on the conduct of overviews of reviews by the Cochrane Collaboration [13].

### Search strategy

In consultation with an experienced librarian, we developed a comprehensive search strategy (see Additional file 1: Appendix 1: Search strategies). We used a systematic review filter developed by the Health Information Research Unit at McMaster University [14], and searched MEDLINE, EMBASE and CINAHL databases to identify systematic reviews published in the past 10 years through to March 30, 2021. We also screened the reference lists of all eligible SRMAs.

### Eligibility criteria

To be eligible, systematic reviews needed to: 1) be published in past 10 years, 2) conduct meta-analyses, 3) include primary studies in adults (≥ 18 years of age), 4) investigate the effects of lower versus higher intake of SFA, or replacement of SFA with PUFA, MUFA, carbohydrate or protein, based on dietary fat or foods containing more than 5 g SFA per 100 g (e.g., cheese, butter, red and processed meat) [15, 16], 5) assess SFA intake for the prevention of critically important outcomes reported in observational studies or RCTs (e.g., all-cause mortality, cancer mortality and incidence, cardiovascular mortality, coronary heart disease (CHD), combined cardiovascular events, stroke, myocardial infarction, type 2 diabetes (T2D), health-related quality of life), and 6) assess the certainty of evidence for the outcomes using a formal certainty of evidence instrument (e.g., GRADE, NutriGrade). To control for biases among outcomes of lower clinical relevance, we only included surrogate outcome data reported in systematic reviews of RCTs (e.g., triglycerides, apolipoprotein A1 (ApoA1), apolipoprotein B (ApoB), LDL, high-density lipoprotein (HDL), total cholesterol, systolic blood pressure, diastolic blood pressure, and changes in weight and body mass index (BMI) (PROSPERO CRD42020172141).

### Study selection process

Two reviewers independently and in duplicate screened the titles, abstracts and full-text articles. We resolved any disagreement through discussion and consulted a senior investigator when disagreements could not be resolved.

### Assessment of the quality of conduct of the included systematic reviews

Two reviewers independently assessed the quality of conduct of included SRMAs using a modified version of the AMSTAR-2 instrument [17]. Our modification addressed concerns we perceive as critical in the conduct of SRMAs: 1) reporting of absolute estimates of effect; and 2) the assessment of the overall certainty of evidence for each outcome (e.g., GRADE, NutriGrade). Among a total of 18 items considered, 9 items were considered of higher importance when assessing the quality of conduct of an SRMA. Our critical items required information from eligible SRMAs regarding protocol registration, comprehensiveness of literature search, justification for excluding studies from the review, appropriateness of statistical methods for meta-analysis, risk of bias assessment of included studies, consideration of the risk of bias during the interpretation of the overall results, consideration of the potential impact of publication bias in the review, reporting of absolute effects, and the assessment of the overall certainty of evidence for each outcome. We rated the overall quality of each review as high, moderate, low or critically low. See Additional file 1: Appendix 2 for the guidance used for rating quality of reviews using our modified version of AMSTAR-2.

### Data collection

We extracted information about the SRMAs (e.g., authors, title, number of included studies, publication year), search strategies (e.g., names of databases searched, database search date, date of last search update), population (e.g., number of participants, age, sex, setting), interventions/exposures (e.g., intervention type, dose and frequency, food or nutrient replacement), comparators (e.g., comparator type, dose, frequency, replacement), and outcomes (as described under eligibility).

### Analysis

For each outcome reported in each review, we presented the exposure, comparator, number of studies and participants, the baseline risk, the absolute and relative effects and the corresponding certainty of evidence. We used data from GLOBOCAN [18] and the Emerging Risk Factors Collaboration [19] to estimate the baseline risks for cancer and major cardiometabolic outcomes, respectively. Using these baseline risks, we calculated the absolute risk reductions for our respective outcomes using the relative risks reported in our included meta-analyses [20]. We categorized the magnitude of effects as very small, small but important, moderate, or large using guidance from GRADE and the Cochrane Collaboration [21, 22]. We used thresholds for the magnitudes of effect from a series of SRMAs on red and processed meat to inform dietary recommendations [23], which were based on consultation with a dietary guideline panel, including members of the public. For fatal outcomes, ≤ 10 events per 1000 were considered to be a very small effect size, 11–25 per 1000 were considered small but important and 26–40 per 1000 were considered a moderate effect size. For non-fatal outcomes, ≤ 20 per 1000 were considered very small, 21–40 per 1000 were considered small but important, and 41–60 per 1000 were considered a moderate effect. For mixed fatal and non-fatal outcomes, ≤ 15 per 1000 were considered very small, 16–30 per 1000 were considered small but important, and 31–45 per 1000 were considered moderate in size. For cardiometabolic outcomes, effect sizes were based on 10.8 years follow-up; for cancer, effect sizes were based on a lifetime of follow-up [18, 19]. For health-related quality of life instruments, we used available estimates of the minimal important difference [24]. As per GRADE guidance, we presented our data in summary of findings tables and used plain language recommendations to describe the magnitude of effect and certainty of evidence [20].

Because we included multiple systematic reviews that reported data on the same outcome, we reported the range of absolute risk reductions (ARR) from our included SRMAs. For example, if there were 5 systematic reviews reporting 4, 9, 8, 15 and 20 fewer cases of stroke comparing lower versus higher intake of SFA, we reported a range of 4 to 20 fewer stroke cases. We reported all absolute effects as a risk difference when lower intake was compared to higher intake. When studies reported results from higher versus lower intake comparisons, we inverted the risk ratio before calculating absolute effects. Our summary of findings in the main text focused on reviews judged as high quality based on our methodological assessment using a modified version of AMSTAR-2. We also highlight evidence for small but important effects for outcomes that were statistically significant, regardless of the quality of evidence. Detailed summaries of all studies are found in Table 1 and Additional file 1: Appendix 3 to 5.

**Table 1:** Characteristics of included systematic reviews

**Reference**	**Study design**	**# of studies (N)**	**Exposure(s)/intervention(s)**	**Comparator(s)**	**Outcomes**	^**a**^**Quality of SRMA conduct**
Jakobsen 2021 [25]	Cohort	Cheese: 7 (554,323), butter: 4 (128,757)	Higher intake of cheese and butter	Lower intake of cheese and butter	CHD (fatal and non-fatal), Stroke (fatal and non-fatal)	High
Schwab 2021 [26]	Cohort	5 (22,591)	Higher intake of SFA (dietary fat)	Lower intake of SFA (dietary fat); Replace SFA with PUFA, MUFA and CHO	Cardiovascular mortality in those with T2D	Moderate
Kazemi 2021 [27]	Cohort, case-control	Red meat: 20 (2,025,667), Processed meat: 17 (1,198,664), Dairy: 10 (544,481), Cheese: 10 (1,419,872)	Higher intake of red and processed meat, dairy and cheese	Lower intake of red and processed meat, dairy and cheese	Breast cancer	Moderate
Neuenschwander 2020 [28]	Cohort	11 (317,423)	Lower intake of SFA (dietary fat)	Higher intake of SFA (dietary fat)	T2D	Moderate
Zeraatkar 2019 [29]	Cohort	55 (4,297,443)	Lower intake of red and processed meat	Higher intake of red and processed meat	All-cause mortality, CVD mortality, T2D, MI (fatal and non-fatal), stroke (fatal and non-fatal), combined CVD events	High
Han 2019 [30]	Cohort	118 (6.1 million)	Lower intake of red and processed meat	Higher intake of red and processed meat	Cancer incidence, cancer mortality	High
Bechthold 2019 [31]	Cohort	Dairy: 24 (783,832), red meat: 15 (26,429), processed meat: 14 (23,607)	Higher intake of dairy, red and processed meat	Lower intake of dairy, red and processed meat	Heart failure, fatal and non-fatal CHD and stroke	Moderate
Schwingshackl 2018 [32]	Cohort	Dairy: 18 (1,629,366), red meat: 25 (2,159,736), processed meat: 18 (2,032,372)	Higher intake of dairy, red and processed meat	Lower intake of dairy, red and processed meat	Cancer incidence (colorectal cancer)	Moderate
Schwingshackl 2017 [33]	Cohort	Dairy: 27 (938,817), red meat: 12 (1,762,627), processed meat: 7 (1,217,965)	Higher intake of dairy, red and processed meat	Lower intake of dairy, red and processed meat	All-cause mortality	Moderate
Schwingshackl 2017 [34]	Cohort	Dairy: 9 (116,415); red meat: 9 (264,148); processed meat: 5 (603,020)	Higher intake of dairy, red and processed meat	Lower intake of dairy, red and processed meat	Hypertension	Low
Schwingshackl 2017 [35]	Cohort	Dairy: 21 (566,872); red meat: 15 (586,040); processed meat: 14 (550,342)	Higher intake of dairy, red and processed meat	Lower intake of dairy, red and processed meat	T2D	Moderate
De Souza 2015 [36]	Cohort	41 (1,211,972)	Lower intake of SFA (dietary fat)	Higher intake of SFA (dietary fat)	All-cause mortality, CVD mortality, T2D, CHD (fatal and non-fatal), CHD (fatal), stroke (fatal and non-fatal)	Moderate
Pham 2014 [37]	Cohort, case-control	19 (573,889)	Higher intake of total, red and processed meat	Lower intake of total, red and processed meat	Cancer incidence	Critically low
Hooper 2020 [38]	RCTs	15 (~59,000)	Lower intake of SFA (dietary fat); Replace SFA with PUFA, MUFA, CHO and protein	Higher intake of SFA (dietary fat)	All-cause mortality, CVD mortality, cancer mortality, cancer incidence, T2D, MI (fatal and non-fatal), MI (non-fatal), CHD (fatal and non-fatal), CHD (fatal), stroke (fatal and non-fatal), combined CVD events, TG, LDL-C, HDL-C, TC, SBP, DBP, quality of life, changes in weight, BMI	High
Uusitupa 2019 [39]	RCTs	7 (4,090)	Lower intake of SFA (dietary fat)	Higher intake of SFA (dietary fat)	T2D	Critically low
Zeraatkar 2019 [40]	RCTs	12 (54,764)	Lower intake of red and processed meat	Higher intake of red and processed meat	All-cause mortality, CVD mortality, cancer mortality, fatal stroke	High
Schwingshackl 2014 [41]	RCTs	11 (6,744)	Lower intake of SFA (dietary fat), Replace SFA with PUFA	Higher intake of SFA (dietary fat)	All-cause mortality, CVD mortality, MIs (fatal and non-fatal), combined CVD events	Critically low

*BMI* Body mass index, *CHD* Coronary heart disease, *CHO* Carbohydrate, *CVD* Cardiovascular Disease, *DBP* Diastolic blood pressure, *HDL-C* High density lipoprotein-cholesterol, *LDL-C* Low density lipoprotein-cholesterol, *MI* Myocardial infarction, *MUFA* Monounsaturated fatty acids, *N* Total number of participants, *PUFA* Polyunsaturated fatty acids, *SBP* Systolic blood pressure, *SF* Saturated fat, *SRMA* Systematic review and meta-analysis, *T2D* Type 2 diabetes, *TC* Total cholesterol

^a^Quality of SRMA conduct using modified version of AMSTAR-2

### Patient involvement

We did not involve any patients or public partners in our overview of systematic reviews, but used effect size ranges that were developed in partnership with stakeholders from the public [23].

## Results

**Fig. 1:**
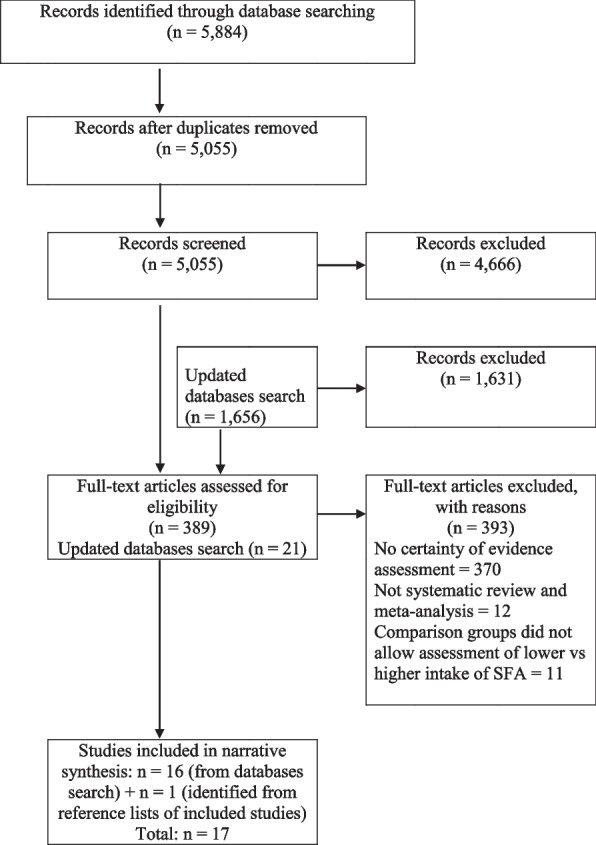
PRISMA flow diagram - summary of study selection

Our literature search yielded 7,540 records. Following screening of the titles and abstracts, we identified 410 potentially eligible SRMAs, of which 17 were eligible after full-text screening (Fig. 1). The included SRMAs were published between 2014 and 2021. Among them, 13 were reviews of observational studies (e.g., cohorts, case-controls) [25–37, 42] and 4 were reviews of RCTs [38–41]. The characteristics of the included SRMAs are reported in Table 1. Based on the modified AMSTAR-2 instrument, the quality of SRMAs was critically low to moderate for 12 reviews (3 critically low, 1 low, 8 moderate) and high for 5 reviews (Additional file 1: Appendix 3). The most frequent limitations related to study selection, conduct and reporting of absolute effect estimates, sources of funding, and heterogeneity exploration via a priori subgroups (Additional file 1: Appendix 3).

The 17 eligible systematic reviews assessed the association of SFA reduction or replacement based on dietary fat [26, 28, 36, 38, 39, 41] or food intake (e.g., cheese, butter, dairy, red meat, processed meat) [25, 27, 29–35, 37, 40, 42] (Additional file 1: Appendix 4 and 5). Among SRMAs of observational studies addressing foods, 9 studies also reported dose-response meta-analysis results based on 120 g, 100 g or 85 g of red meat, 50 g or 30 g of processed meat, and 200 g of dairy intake per day [27–35]. We summarized the results of SRMAs addressing foods as lower versus higher intake of red meat, processed meat, cheese, butter, and dairy. Among the 4 SRMAs of RCTs reporting on SFA reduction, 2 reviews also reported subgroups or meta-regression to explore effect modification including replacing SFA with PUFA, MUFA, carbohydrate, or protein [38, 41], as well as percentage of baseline energy intake from SFA, percentage of difference in energy intake from SFA, total cholesterol and sex [38] (Additional file 1: Appendix 6 to 13). Results are reported below for the higher quality SRMAs, their corresponding estimates of effect including any evidence of small but important statistically significant effects, the certainty of the estimates and relevant subgroup/meta-regression effects.

### All-cause mortality

Six SRMAs reported on all-cause mortality associated with SFA reduction or replacement, including 3 reviews of observational studies (follow-up ranging from 1 to 32 years) [29, 33, 36] and 3 reviews of RCTs (follow-up ranging from 6 months to 17 years) [38, 40, 41]. The quality of reviews ranged from critically low to high quality. Two high quality SRMAs of RCTs on dietary fat and processed meat [38, 40] and 1 high quality review of observational studies of red meat [29] suggested very small absolute effects ranging from 1 to 9 fewer deaths per 1000 people based on very low to moderate certainty of evidence (CoE). One moderate quality SRMA of observational studies [33] showed a small but important statistically significant ARR of 20 fewer events per 1000 people based on moderate CoE for lower versus higher processed meat intake [33] (Additional file 1: Appendix 3 and 4).

### Cancer mortality

Three SRMAs reported on cancer mortality associated with reducing or replacing SFA, including 1 review of observational studies (follow-up ranging from 3 to 34 years) [30], and 2 reviews of RCTs (follow-up ranging from 6 months to 17 years) [38, 40]. All reviews were judged as methodologically high quality. The SRMA of observational studies on red and processed meat [30] was of high quality, suggesting a very small ARR of 8 fewer to 3 more events per 1000 people followed based on very low to low CoE for gastric, colorectal, pancreatic, prostate and overall cancer mortality. Similarly, the two SRMAs of RCTs on dietary fat and red meat intake were of high quality [38, 40], suggesting no to small but important effects ranging from zero events to 12 fewer events per 1000 people based on very low and unreported CoE (Additional file 1: Appendix 3 and 4).

### Cancer incidence

Five SRMAs reported on cancer incidence associated with SFA reduction or replacement, including 4 reviews of observational studies (follow-up ranging from 2 to 34 years) [27, 30, 32, 37] and 1 review of RCTs [38] (follow-up ranging from 4 to 5 years on average). The quality of reporting of reviews ranged from critically low to high quality. One SRMA of observational studies assessing red and processed meat intake was high quality [30] suggesting a very small ARR of 2 to 13 fewer overall cancer cases per 1000 people followed based on very low CoE. This study also suggested very small to no absolute effects with specific cancers, including small intestinal (0 fewer events, low CoE), pancreatic (0 fewer events, low CoE), oral (1 fewer event, very low CoE), endometrial (1 fewer event, very low CoE), ovarian (1 fewer event, low CoE), hepatic (1 more event, very low CoE), esophageal (0 to 2 fewer events, very low CoE), gastric (2 fewer events, very low CoE), breast (5 to 6 fewer events, low CoE), prostate (0 to 1 more event, low CoE), and colorectal (0 to 1 fewer events, low CoE) cancer per 1000 people. The 1 review of RCTs that investigated dietary fat specifically and was rated high quality [38] suggested a very small ARR on overall cancer incidence of 11 fewer events per 1000 people without reporting on the CoE (Additional file 1: Appendix 3 and 4).

### Cardiovascular mortality

Five SRMAs reported on cardiovascular mortality associated with reducing or replacing SFA, including 2 reviews of observational studies (follow-up ranging from 1 to 32 years) [29, 36] and 3 reviews of RCTs (follow-up ranging from 6 months to 17 years) [38, 40, 41]. The reporting of reviews ranged from critically low to high quality. One SRMA of observational studies assessing red and processed meat was high quality [29] and suggested a very small ARR of 4 fewer events per 1000 people followed based on very low CoE. Two of the three SRMAs of RCTs assessing dietary fat and red meat were of high quality [38, 40] suggesting a very small ARR of 2 to 3 fewer events per 1000 people based on very low and moderate CoE, respectively. For replacing SFA with PUFA and carbohydrate, one moderate quality SRMA of cohort studies reported the risk of cardiovascular mortality in participants with type 2 diabetes [26]. Schwab et al. [26] reported a small but important statistically significant effect of 15 fewer cardiovascular deaths per 1000 individuals with type 2 diabetes followed when replacing 2% total energy SFA with PUFA (95%CI 26 to 1 fewer) based on very low CoE. Authors also reported a small but important effect that was almost statistically significant, of 20 fewer cardiovascular deaths per 1000 (95%CI 37 to 0 fewer) when replacing 5% total energy from SFA with higher fiber CHO, which was also based on very low CoE [26] (Additional file 1: Appendix 3 and 4).

### Coronary heart disease 

Four SRMAs reported on CHD associated with SFA reduction or replacement, including 3 reviews of observational studies (follow-up ranging from 1 to 32 years) [25, 31, 36] and 1 review of RCTs (follow-up ranging from 4 to 5 years, on average) [38]. The reporting of reviews ranged from moderate to high quality. One SRMA of observational studies investigating dairy (butter, cheese) intake was high quality [25] and suggested a very small ARR for fatal and non-fatal CHD of 0 to 4 fewer events per 1000 people followed based on low and moderate CoE. The 1 SRMA of RCTs investigating dietary fat intake was high quality, suggesting 8 fewer fatal and non-fatal events and 1 fewer fatal event per 1000 people based on very low and low CoE, respectively (Additional file 1: Appendix 3 and 4).

### Combined cardiovascular events

One SRMA reported on combined cardiovascular events. The review was high quality, evaluated RCT evidence, and measured SFA reduction or replacement [38]. Authors reported a small but important statistically significant effect (16 fewer absolute cardiovascular events) per 1000 individuals followed (Additional file 1: Appendix 4) based on moderate CoE.

### Stroke

Six SRMAs reported on stroke (fatal and non-fatal) associated with reducing or replacing SFA, including 4 reviews of observational studies (follow-up ranging from 1 to 32 years) [25, 29, 31, 36] and 2 reviews of RCTs [38, 40] (follow-up ranging from 6 months to 17 years). The reporting of reviews ranged from moderate to high quality. Two of the SRMAs of observational studies investigating red and processed meat intake and diary intake were high quality [25, 29], suggesting a very small ARR of 1 fewer to 2 more events per 1000 people followed based on very low to moderate CoE (Additional file 1: Appendix 3 and 4).

### Myocardial infarction

Three SRMAs reported on myocardial infarction associated with SFA reduction or replacement, including 1 review of observational studies (follow-up ranging from 2 to 28 years) [29] and 2 reviews of RCTs (follow-up ranging from 1 to 17 years) [38, 41]. The reporting of reviews ranged from critically low to high quality. The SRMA [29] of observational studies investigating red and processed meat was high quality and suggests a very small ARR of 2 to 3 fewer events per 1000 people followed based on very low CoE. One SRMA of RCTs investigating SFA intake was high quality [38], suggesting a very small ARR of 1 to 4 fewer events per 1000 people based on very low to low CoE (Additional file 1: Appendix 3 and 4).

### T2D

Six SRMAs reported on T2D associated with SFA reduction or replacement, including 4 reviews of observational studies (follow-up ranging from 1 to 32 years) [28, 29, 35, 36] and 2 reviews of RCTs (follow-up ranging from 1 to 6 years) [38, 39]. The reporting of reviews ranged from moderate to high quality. One of the SRMAs of observational studies investigating red and processed meat was high quality [29] and suggested a very small ARR on T2D incidence ranging from 6 to 12 fewer events per 1000 people followed based on low to very low CoE. One SRMA of RCTs investigating dietary fat intake was high quality [38] and suggested a very small ARR of 2 fewer events per 1000 people without a reported CoE (Additional file 1: Appendix 3 and 4).

### Health-related quality of life

One SRMA reported on health-related quality of life based on a single RCT that evaluated a reduced fat diet from the Woman’s Health Initiative trial [38]. This review was high quality, evaluated RCT evidence and measured dietary SFA; data on CoE were not reported. At ‘close out’ (~8 years post randomization), the intervention diet demonstrated a beneficial effect of lower SFA intake as measured using a global question regarding health-related quality of life (“Overall, how would you rate your quality of life?”; scale: 0 [worst] to 10 [best]). While participants experienced an improvement (MD 0.04, 95% CI 0.01 to 0.07), the improvement was very small (Additional file 1: Appendix 3 and 4).

### Surrogate outcomes

One high quality SRMA of RCTs reported on surrogate outcomes at 2 years [38], demonstrating beneficial effects of SFA reduction or replacement on LDL (MD -0.19 mmol/L, 95% CI -0.33 to -0.05), total cholesterol (MD -0.24 mmol/L, 95% CI -0.36 to -0.13), and BMI (MD -0.42, 95% CI -0.72, -0.12) (Additional file 1: Appendix 5). Based on estimated minimal clinically important differences, the estimate for LDL was almost twice the important difference (0.10 mmol/L), while the estimate for total cholesterol was slightly below the important difference (0.26 mmol/L) [43]. We are unaware of the minimal clinically important difference for BMI.

### Subgroup analysis

Regarding subgroup effect modification, Hooper et al. [38] reported statistically significant tests of interaction for percentage of baseline energy intake from SFA with higher baseline intake (i.e., from up to 12% energy from SFA at baseline to > 18% energy from SFA at baseline) being associated with higher combined cardiovascular events (range, 2 to 45 fewer cases of combined cardiovascular events per 1000). Similarly, a subgroup on percent difference in energy intake from SFA (i.e., up to 4% energy difference, > 4 to 8% energy difference, > 8% energy difference) showed that larger reductions in SFA were associated with fewer combined cardiovascular events (range: 2 to 32 fewer cases of combined cardiovascular events per 1000), although the relationship was not linear. Subgroups based on biological sex showed statistically significant effects of lowering SFA intake in males as well as total cholesterol reduction (at least 0.2 mmol/L, < 0.2 mmol/L) for reducing the risk of combined cardiovascular events. The between group analysis for replacement of SFA with PUFA, MUFA, carbohydrate and protein did not show statistically significant tests of interaction, while within group analyses showed statistically significant results based on the 7 of 12 trials that replaced SFA with PUFA demonstrated a small but important ARR (21 fewer combined CVD events per 1000). For CHD, Hooper et al. [38] reported a very small effect that was close to statistically significant (11 fewer; 45 to 0 fewer events) (Additional file 1: Appendix 3, 4, 13).

Apart from combined cardiovascular events and CHD [38], the subgroup and meta-regression analysis for each of the effect modifiers (e.g., replacement of SFA with PUFA, MUFA, carbohydrate, or protein; percentage of baseline energy intake from SFA; percentage of difference in energy intake from SFA; total cholesterol and sex) did not show statistically significant tests of interaction for all-cause mortality, cardiovascular mortality, stroke, myocardial infarction, T2D or any of the surrogate outcomes [38, 41] (Additional file 1: Appendix 6 to 13).

## Discussion

### Summary of main results

In total, we included 17 SRMAs assessing the impact of reducing saturated fat or fatty foods (cheese, butter, dairy, red meat, and processed meat), or replacing saturated fat with PUFA, MUFA, carbohydrates or protein, on the risk of mortality and major cancer and cardiometabolic outcomes. Among the included studies, the quality of the SRMAs was critically low to moderate for 12 reviews (3 critically low, 1 low, 8 moderate) and high for 5 reviews using a modified version of AMSTAR-2 instrument.

Among 5 high quality SRMAs, 2 were reviews of RCTs and 3 were reviews of observational studies. The 3 SRMAs of observational studies almost universally reported low or very low certainty evidence for a very small association of SFA intake and the risk of cancer and cardiovascular outcomes [26, 29, 30]. Among our 2 high quality reviews of RCTs [38, 40], in one review on SFA reduction or replacement, we found moderate certainty evidence for a statistically significant decreased risk of combined cardiovascular events (16 fewer events) per 1000 people [38]. The effect of reducing or replacing SFA intake for the remaining outcomes among all high-quality reviews was very small based predominantly on low and very low CoE. Among 8 moderate quality SRMAs, two reviews showed small but important effects. Among these 8 reviews, Schwingshackl et al. [33] reported a statistically significant effect for a lower risk of all-cause mortality (20 fewer events per 1000 followed) among those with lower processed meat intake based on moderate certainty evidence. Schwab et al. [26] reported a small but important statistically significant effect of 15 fewer cardiovascular deaths per 1000 individuals with type 2 diabetes followed when replacing 2% total energy SFA with PUFA (95%CI 26 to 1 fewer) based on very low CoE. The effect of reducing or replacing SFA intake for the remaining outcomes among all moderate quality reviews was very small based predominantly on moderate CoE.

Based on diverse approaches to rating the certainty of evidence including GRADE, NutriGrade and guidance from WHO/FAO Expert Consultation Report, the CoE across all outcomes was typically low to very low, but ranged from very low to high. In many instances, the CoE may have been overrated, which may be due to overlooking issues related to risk of bias and indirectness. For example, the use of self-reported dietary instruments to assess dietary intakes increase the risk of bias due to poor recall or social desirability [44, 45]. Further, many nutrition studies suffer from excessive missing participant outcome data for which a simple intention-to-treat analysis does not adequately address the risk of bias [46]. Finally, complex interventions or exposures including the combined effects of dietary patterns present serious indirectness issues when, for instance, attempting to explore the specific effects of dietary SFA in isolation [39]. Indirectness issues are further compounded by the fact that foods high in SFA also contain important nutrients including other essential fats such as PUFA and MUFA. For example, red meat is high in SFA, but also rich in vitamins A, B12, D and K, as well as iron, selenium, zinc, MUFA and PUFA [47]. Further, based on high quality systematic reviews, while certain sources of saturated fat, such as dairy products, may have very small cardiovascular protective effects based on low to moderate certainty evidence [25] others, like unprocessed red meat, may slightly increase the risk of cardiovascular disease based on low or very low certainty evidence [30, 40]. It is also important to note that the context in which saturated fat is consumed can also play a role on its potential impact on cardiovascular health. For example, studies have shown that when saturated fat is consumed within the context of a healthy overall dietary pattern, potential adverse cardiovascular events may be attenuated [48]. Overall, regardless of the source of saturated fat, the effects are typically very small (< 1%) and not distinguishable. Moreover, the certainty of the evidence is predominantly very low to low, which further adds to our inability to discern between the various sources of saturated fat and the impact on critically important outcomes like major cardiovascular events or cancer.

### Results in context to previous reviews of saturated fat

A recent scoping review of systematic reviews of observational studies and RCTs by Schwingshackl et al. [49] assessed the effects of total dietary fat and fat quality (e.g., SFA, MUFA, PUFA, trans-fatty acid) on all-cause mortality, cancer, chronic disease outcomes (e.g., CVD, CHD, stroke, T2D), weight, and surrogate outcomes (e.g., cholesterol, blood pressure, fat mass, waist circumference) in adults. The scoping review included 59 reviews for descriptive synthesis. While our overview included reviews that assessed the certainty of evidence as well as a method to document the absolute magnitude effect, among the 59 reviews in Schwingshackl et al. [49], authors only reported relative effects and very few (7/59; 12%) included reviews assessed the certainty of evidence for each outcome reported. Further, while Schwingshakl et al. reported results similar to ours, their scoping review did not assess the quality of included reviews, a strength of our study.

Similarly, a second recent narrative review of systematic reviews, observational studies and RCTs by Astrup et al. [12] addressing SFA and health outcomes indicated that systematic reviews did not find a significant association between SFA reduction or replacement of SFA with PUFA and all-cause mortality. Unlike our overview, authors did not assess or report the methodological quality of included reviews, or the absolute estimates of effect for each outcome, including the certainty of the estimates.

### Limitations of our study methods

Our review was not without limitations. First, we only included SRMAs that assessed the CoE using a formalized approach such as GRADE or NutriGrade. While this may have limited inclusion of some higher quality reviews that did not assess CoE [50], in recognizing that international standards for SRMAs and the call for presenting absolute effect estimates together with the CoE [23, 51–53], we opted to present the absolute magnitudes of effect exclusively in the context of the certainty of effect estimates for our target outcomes. Second, rather than re-assessing the CoE ourselves for each outcome within each SRMA, we relied on assessments from the authors of the included SRMAs. This introduced some heterogeneity as the CoE method (e.g., GRADE, NutriGrade) differed among reviews. For example, discrepant CoE results have been demonstrated when applying GRADE versus NutriGrade to the same systematic summary evidence [43]. That is, the application of GRADE, a rigorous approach based on over 30 published guidance papers and formally adopted by over 100 authoritative organizations worldwide (e.g. Cochrane Nutrition, WHO) [54, 55], tends to conservatively rate the CoE lower than NutriGrade methods [43]. Among our 5 high quality reviews, 4 used GRADE, which is more robust, to rate the CoE while one used NutriGrade [25]. Third, while we determined the absolute magnitude of effect (very small, small, moderate, large) for fatal and non-fatal outcomes over 10.8 years (cardiometabolic) to a lifetime (cancer) based on thresholds used in a high quality dietary guideline addressing red and processed meat [23], there is no general consensus based on surveys of members of the public for threshold estimates specific to mortality and major cancer and cardiometabolic outcomes (e.g., stroke, myocardial infarction). Rather, these thresholds are based on consensus among 18 guideline panel members including clinicians, scientists and members of the public across 7 countries. The thresholds considered trade-offs across all outcomes important to decision-making (e.g., mortality, health-related quality of life, and dietary satisfaction). Fourth, in calculating absolute effects for cardiovascular outcomes, we used data from the Emerging Risk Factors Collaboration that includes data from over 100 studies with well-defined criteria and 8.5 million person-years at risk. However, this dataset pools from studies that include participants without initial vascular disease [19]. In cases of secondary cardiovascular disease prevention, we would expect larger absolute effect sizes than we calculated using the Emerging Risk Factors Collaboration. As a result, our estimates should be considered most applicable to the primary prevention of cardiovascular disease, and considered in the context of national dietary guidelines that focus on primary prevention [5]. Individuals at higher cardiovascular risk typically have access to effective lipid-lowering drugs (statins) [56], and the effectiveness of modified SFA in addition to drugs is generally unknown [38]. Finally, this project was in-part funded by Texas A&M AgriLife. As a result, some readers may believe there is a bias in favor of animal-based foods. The AgriLife funds were for investigator-initiated research related to saturated and polyunsaturated fats, and from interest and investment earnings, not a sponsoring organization, industry, or company. Further, it must be noted that the corresponding authors affiliations with NutriRECS and EBN.org endeavors to apply GRADE, Guidelines International Network, Cochrane and Joanna Briggs Institute endorsed methods to evidence synthesis allowing readers to have clear summaries of the best available evidence, methods that include absolute estimates and the certainty of estimates. While to our knowledge no national dietary guidelines adhere to these methods, these methods make decision-makers vividly aware of the magnitude of effect and certainty of evidence for all health outcomes, ideally based on high quality SRMAs only.

### Strengths of our study methods and findings

Our review has several strengths. First, we conducted this review following published Cochrane guidance on the conduct of overviews of reviews [13]. Two reviewers independently screened, selected, and extracted data including the estimates of effect and the corresponding CoE for each outcome, and assessed the quality of conduct of each SRMA using a modified version of AMSTAR-2. Second, we utilized the AMSTAR-2 critical appraisal instrument instead of the risk of bias in systematic reviews (ROBIS) instrument to assess the quality of the included SRMAs. AMSTAR-2 has slightly higher reliability compared to ROBIS [57] and provides instructions that are very easy to apply. Regarding our modifications of AMSTAR-2, we included two additional quality items based on a recent systematic survey of review methodology [58]. In particular, based on Cochrane guidance, we believe assessing CoE is necessary to help ensure the validity and interpretability of SRMAs on an outcome by outcome basis [59]. Also, based on Cochrane guidance, we added an item on the reporting of absolute estimates of effect. Formal Cochrane guidance has highlighted concerns with the exclusive use of relative effects for dichotomous outcome [55]. When the same treatment effects are expressed in both relative and absolute terms, relative effects often yield apparently larger estimates as compared to absolute effects (e.g., in moving from a 2% risk in the control group to 1% in the exposure groups, one arrives at 50% relative risk reduction versus 1% absolute risk reduction) [60, 61]. To avoid misleading clinicians, patients and members of the public, and to balance the benefits and harms of an intervention/exposure, authors of SRMAs should provide review users with absolute effect estimates [62]. While GRADE and Cochrane guidance support the use of absolute effects in summary of findings tables of SRMAs [20, 53, 63], a recent systematic survey of 150 non-Cochrane systematic reviews in the field of nutrition reported that only 5 (3.5%) of 150 reviews in nutrition reported absolute effects [64]. Third, as mentioned above, we modified AMSTAR-2 including two additional items and we bolstered the criteria for item 14 on the assessment of between-study heterogeneity. Although these modifications to AMSTAR-2 have not undergone a formal consensus and validation, we believe they are justified given the importance of reporting CoE [59], absolute effects [62] and reporting a priori subgroup analysis plans [65].

### Implications for practice

Our findings show considerable uncertainty around linking SFA reduction with improved health outcomes. These findings are contrary to the orthodox position of the nutrition community wherein the majority of national nutrition guidelines promote reducing or replacing SFA intake for the general population [5, 66]. Across our 17 eligible SRMAs that reported over 100 outcomes (Additional file 1: Appendix 4), only three outcomes showed moderate to high CoE for a small but important effect that was statistically significant – all-cause mortality [33], combined CV events [38], and mortality in patients with T2D [39]. In contrast, 5 reviews showed very small effects on all-cause mortality, with four reporting effects that were not statistically significant [29, 36, 38, 40, 41]. Given the conflicting results for all-cause mortality and the limitations of combining cardiovascular outcomes of varying importance to patients [67], health professionals should ideally share the small and uncertain estimates of effect with patients to encourage informed, value and preference sensitive decision-making.

### Implications for future research

Among 17 included SRMAs, the quality of the reviews was critically low to moderate for 12 reviews (3 critically low, 1 low, 8 moderate) and high for 5 reviews. Based on the most frequent limitations across our SRMAs included in our overview of reviews, future reviews should report absolute effect estimates and conduct robust heterogeneity explorations using a priori subgroup analysis reported in publicly available SRMA study protocols. In addition, rather than assessing the certainty of evidence for a non-null effect (*P* > 0.05), as we have done, investigators conducting reviews should use GRADE guidance to contextualize the absolute treatment or exposure effects using thresholds for small but important effects [22]. Improved reporting quality will make reviews more interpretable for members of the public, patients, clinicians and policy-makers. Given most individuals at moderate to high cardiovascular risk would be administered effective lipid-lowering drugs (e.g., statins), long-term future trials should focus on reducing SFA or replacing SFA with PUFA in lower risk individuals using valid biomarkers for baseline and achieved (post-intervention) fatty acid levels [68].

## Conclusions

Systematic reviews investigating the impact of SFA on mortality and major cancer and cardiometabolic outcomes almost universally suggest very small absolute changes in risk, and the data is based primarily on low and very low certainty evidence.

### Supplementary Information

**Additional file 1: Appendix 1.** Search strategies. **Appendix 2.** The guidance for rating quality of reviews using modified version of AMSTAR-2 instrument. **Appendix 3.** Quality of conduct of included systematic reviews based on modified AMSTAR-2 instrument. **Appendix 4.** Summary of findings of systematic reviews of observational studies and randomized controlled trials. **Appendix 5.** Summary of findings of surrogate outcomes. **Appendix 6.** Subgroup analysis - all-cause mortality (Hooper et al. 2020) [38]. **Appendix 7.** Subgroup analysis – cardiovascular mortality (Hooper et al. 2020) [38]. **Appendix 8.** Subgroup analysis - myocardial infarction (Hooper et al. 2020) [38]. **Appendix 9.** Subgroup analysis - myocardial infarction (non-fatal) (Hooper et al. 2020) [38]. **Appendix 10.** Subgroup analysis - coronary heart disease (fatal and non-fatal) (Hooper et al. 2020) [38]. **Appendix 11.** Subgroup analysis - coronary heart disease (fatal) (Hooper et al. 2020) [38]. **Appendix 12.** Subgroup analysis - stroke (fatal and non-fatal) (Hooper et al. 2020) [38]. **Appendix 13.** Subgroup analysis - combined cardiovascular events (Hooper et al. 2020) [38].

## Data Availability

No additional data available.

## Abbreviations

AMSTAR-2: A measurement instrument to assess systematic reviews
ARR: Absolute risk reduction
BMI: Body mass index
CHD: Coronary heart disease
CHO: Carbohydrate
CI: Confidence interval
CMD: Cardiometabolic disease
CoE: Certainty of evidence
CVD: Cardiovascular disease
DASH: The Dietary Approaches to Stop Hypertension
GLOBOCAN: The Global Cancer Observatory
GRADE: The Grading of Recommendations, Assessment, Development and Evaluation
HDL: High-density lipoprotein
JACC: Journals of the American College of Cardiology
LDL: Low-density lipoprotein
MD: Mean difference
MI: Myocardial infarction
MID: Minimally important difference
MUFA: Monounsaturated fatty acids
NutriGrade: A Scoring System to Assess and Judge the Meta-Evidence of Randomized Controlled Trials and Cohort Studies in Nutrition Research
PUFA: Polyunsaturated fatty acids
RCT: Randomized control trial
ROBIS: An instrument to assess risk of bias in systematic reviews
SFA: Saturated fatty acid
SRMA: Systematic review and meta-analysis
