# Supplementary material for: Saturated fat, the estimated absolute risk and certainty of risk for mortality and major cancer and cardiometabolic outcomes: an overview of systematic reviews
Source: Syst Rev. 2023 Sep 30;12:179. doi: 10.1186/s13643-023-02312-3 (PMC10541715; doi:10.1186/s13643-023-02312-3)
Supplement: Supplementary file 1 — Additional file 1: Appendix 1. Search strategies. Appendix 2. The guidance for rating quality of reviews using modified version of AMSTAR-2 instrument. Appendix 3. Quality of conduct of included systematic reviews based on modified AMSTAR-2 instrument. Appendix 4. Summary of findings of systematic reviews of observational studies and randomized controlled trials. Appendix 5. Summary of findings of surrogate outcomes. Appendix 6. Subgroup analysis - all-cause mortality (Hooper et al. 2020) [38]. Appendix 7. Subgroup analysis – cardiovascular mortality (Hooper et al. 2020) [38]. Appendix 8. Subgroup analysis - myocardial infarction (Hooper et al. 2020) [38]. Appendix 9. Subgroup analysis - myocardial infarction (non-fatal) (Hooper et al. 2020) [38]. Appendix 10. Subgroup analysis - coronary heart disease (fatal and non-fatal) (Hooper et al. 2020) [38]. Appendix 11. Subgroup analysis - coronary heart disease (fatal) (Hooper et al. 2020) [38]. Appendix 12. Subgroup analysis - stroke (fatal and non-fatal) (Hooper et al. 2020) [38]. Appendix 13. Subgroup analysis - combined cardiovascular events (Hooper et al. 2020) [38]. [file 13643_2023_2312_MOESM1_ESM.docx]

# Supplementary appendices

## Appendix 1: Search strategies

**Database: MEDLINE**

Search Strategy:

--------------------------------------------------------------------------------

1 Apolipoproteins B/

2 Apolipoproteins A/

3 Triglycerides/

4 diet* pattern*.ti,ab.

5 Fatty Acids/

6 fatty acid*.ti,ab.

7 exp Dietary Fats/

8 (saturated adj2 fat*).ti,ab.

9 Food/

10 exp Dairy Products/

11 exp Eggs/

12 exp Meat/

13 Plant Oils/

14 (palm adj2 (oil or oils)).ti,ab.

15 (butter or milk* or cheese* or cream* or meat*).ti,ab.

16 (lamb or pork or poultry or lard).ti,ab.

17 or/1-16

18 (MEDLINE or systematic review).tw. or meta analysis.pt.

19 exp Neoplasms/

20 cardiovascular diseases/ or exp heart diseases/ or exp vascular diseases/

21 cerebrovascular disorders/ or exp brain ischemia/ or exp carotid artery diseases/ or exp dementia, vascular/ or exp intracranial arterial diseases/ or exp "intracranial embolism and thrombosis"/ or exp intracranial hemorrhages/ or exp stroke/

22 (coronar* adj5 (bypas* or graft* or disease* or event*)).ti,ab.

23 (cerebrovasc* or cardiovasc* or mortal* or angina* or stroke or strokes).ti,ab.

24 (myocardi* adj5 (infarct* or revascular* or ischaemi* or ischemi*)).ti,ab.

25 (morbid* adj5 (heart* or coronar* or ischaem* or ischem* or myocard*)).ti,ab.

26 (vascular* adj5 (peripheral* or disease* or complication*)).ti,ab.

27 (heart* adj5 (disease* or attack* or bypass*)).ti,ab.

28 or/19-27

29 17 and 18 and 28 (1697)

30 limit 29 to yr="2009 -Current"

**Database: Embase**

Search Strategy:

--------------------------------------------------------------------------------

1 apolipoprotein B/

2 apolipoprotein A1/

3 triacylglycerol/

4 diet* pattern*.ti,ab.

5 fatty acid/

6 fatty acid*.ti,ab.

7 exp fat intake/ [exp Dietary Fats]

8 (saturated adj2 fat*).ti,ab.

9 food/

10 exp dairy product/

11 exp egg/

12 exp meat/

13 vegetable oil/ [plants oils]

14 (palm adj2 (oil or oils)).ti,ab.

15 (butter or milk* or cheese* or cream* or meat*).ti,ab.

16 (lamb or pork or poultry or lard).ti,ab.

17 or/1-16

18 (meta-analysis or systematic review).tw.

19 exp malignant neoplasm/

20 cardiovascular disease/ or exp heart disease/ or exp vascular disease/

21 cerebrovascular disorders/ or exp brain ischemia/ or exp carotid artery diseases/ or exp dementia, vascular/ or exp intracranial arterial diseases/ or exp "intracranial embolism and thrombosis"/ or exp intracranial hemorrhages/ or exp stroke/

22 (coronar* adj5 (bypas* or graft* or disease* or event*)).ti,ab.

23 (cerebrovasc* or cardiovasc* or mortal* or angina* or stroke or strokes).ti,ab.

24 (myocardi* adj5 (infarct* or revascular* or ischaemi* or ischemi*)).ti,ab.

25 (morbid* adj5 (heart* or coronar* or ischaem* or ischem* or myocard*)).ti,ab.

26 (vascular* adj5 (peripheral* or disease* or complication*)).ti,ab.

27 (heart* adj5 (disease* or attack* or bypass*)).ti,ab.

28 or/19-27

29 17 and 18 and 28

30 limit 29 to yr="2009 -Current"

**Database: CINAHL**

Search strategy

| **#** | **Query** |
| --- | --- |
| S1 | (MH "Apolipoproteins") |
| S2 | (MH "Triglycerides") |
| S3 | TI diet* pattern* OR AB diet* pattern* |
| S4 | (MH "Fatty Acids") |
| S5 | TI fatty acid* OR AB fatty acid* |
| S6 | (MH "Dietary Fats+") |
| S7 | TI saturated N2 fat* OR AB saturated N2 fat* |
| S8 | (MH "Food") |
| S9 | (MH "Dairy Products+") |
| S10 | (MH "Eggs") |
| S11 | (MH "Meat+") |
| S12 | (MH "Plant Oils") |
| S13 | TI ( palm N2 (oil or oils) ) OR AB ( palm N2 (oil or oils) ) |
| S14 | TI ( butter or milk* or cheese* or cream* or meat* ) OR AB ( butter or milk* or cheese* or cream* or meat* ) |
| S15 | TI ( lamb or pork or poultry or lard ) OR AB ( lamb or pork or poultry or lard ) |
| S16 | S1 OR S2 OR S3 OR S4 OR S5 OR S6 OR S7 OR S8 OR S9 OR S10 OR S11 OR S12 OR S13 OR S14 OR S15 |
| S17 | TX meta-analysis |
| S18 | PT review |
| S19 | PT systematic review |
| S20 | S17 OR S18 OR S19 |
| S21 | (MH "Neoplasms+") |
| S22 | (MH "Cardiovascular Diseases") OR (MH "Heart Diseases+") OR (MH "Vascular Diseases+") |
| S23 | (MH "Cerebrovascular Disorders") OR (MH "Cerebral Ischemia+") OR (MH "Carotid Artery Diseases+") OR (MH "Dementia, Vascular+") OR (MH "Intracranial Arterial Diseases+") OR (MH "Intracranial Embolism and Thrombosis+") OR (MH "Intracranial Hemorrhage+") OR (MH "Stroke+") |
| S24 | TI ( coronar* N5 (bypas* or graft* or disease* or event*) ) OR AB ( coronar* N5 (bypas* or graft* or disease* or event*) ) |
| S25 | TI ( cerebrovasc* or cardiovasc* or mortal* or angina* or stroke or strokes ) OR AB ( cerebrovasc* or cardiovasc* or mortal* or angina* or stroke or strokes ) |
| S26 | TI ( myocardi* N5 (infarct* or revascular* or ischaemi* or ischemi* ) OR AB ( myocardi* N5 (infarct* or revascular* or ischaemi* or ischemi* ) |
| S27 | TI ( morbid* N5 (heart* or coronar* or ischaem* or ischem* or myocard* ) OR AB ( morbid* N5 (heart* or coronar* or ischaem* or ischem* or myocard* ) |
| S28 | TI ( vascular* N5 (peripheral* or disease* or complication* ) OR AB ( vascular* N5 (peripheral* or disease* or complication* ) |
| S29 | TI ( heart* N5 (disease* or attack* or bypass*) ) OR AB ( heart* N5 (disease* or attack* or bypass*) ) |
| S30 | S21 OR S22 OR S23 OR S24 OR S25 OR S26 OR S27 OR S28 OR S29 |
| S31 | S16 AND S20 AND S30 |
| S32 | S16 AND S20 AND S30 |

## Appendix 2: The guidance for rating quality of reviews using modified version of AMSTAR-2 instrument

| **High** - if there is no more than one critical weakness, or no more than two non-critical weaknesses |
| --- |
| **Moderate** - if there are two critical weaknesses or three non-critical weakness |
| **Low** - if there are three critical weaknesses or four non-critical weaknesses |
| **Critically low** - if there are four critical weaknesses or five or more non-critical weaknesses |
|  |

Note: For each item, as with AMSTAR-2, we rated each question as “yes” (complete adherence to the standard), “partial yes” (partial adherence to the standard), “no” (non-adherence to the standard). If a SRMA did not follow a specific criterion completely then we marked it as “partial yes” instead of “yes”. If a SRMA had two “partial yes” judgments, we considered these as equal to one “no”. If a SRMA had two “no” responses to non-critical items, we considered these as a critical item “no”.

## Appendix 3: Quality of conduct of included systematic reviews based on modified AMSTAR-2 instrument

| AMSTAR-2 criteria | Zeraatkar 2019a | Han 2019 | Schwingshackl 2017a | Schwab 2021 | Bechthold 2019 | Jakobsen 2021 | Schwingshackl 2018 | Neuenschwander 2020 | Schwingshackl 2017c |
| --- | --- | --- | --- | --- | --- | --- | --- | --- | --- |
| PICO | Yes | Yes | Yes | Yes | Yes | Yes | Yes | Yes | Yes |
| A priori methods | Yes | Yes | Yes | No | Yes | Yes | Yes | Yes | Yes |
| Inclusion | Yes | Yes | Yes | Yes | Yes | Yes | No | Yes | No |
| Search strategy | Yes | Yes | Yes | Yes | Yes | Yes | Yes | Yes | Yes |
| Study selection | Yes | Yes | Yes | Yes | Yes | Yes | Yes | Yes | Yes |
| Data extraction | Yes | Yes | No | Yes | Yes | No | Yes | No | Yes |
| Study exclusion | PY | PY | PY | PY | PY | Yes | PY | Yes | PY |
| Study characteristics | Yes | Yes | Yes | Yes | Yes | Yes | Yes | Yes | Yes |
| Risk of bias | Yes | Yes | Yes | Yes | Yes | Yes | Yes | Yes | Yes |
| Sources of funding | No | Yes | PY | No | No | Yes | PY | No | PY |
| Meta-analysis methods | Yes | Yes | Yes | Yes | Yes | Yes | Yes | Yes | Yes |
| Impact of risk of bias | Yes | Yes | Yes | Yes | Yes | Yes | Yes | Yes | Yes |
| Account for risk of bias | Yes | Yes | Yes | Yes | Yes | Yes | Yes | Yes | Yes |
| Heterogeneity and a-priori subgroup | Yes | Yes | Yes | No | Yes | Yes | Yes | Yes | Yes |
| Publication bias | Yes | Yes | Yes | Yes | Yes | Yes | Yes | Yes | Yes |
| Conflict of interest | Yes | Yes | Yes | Yes | Yes | Yes | Yes | Yes | Yes |
| Certainty of evidence | Yes | Yes | Yes | Yes | Yes | Yes | Yes | Yes | Yes |
| Absolute effect estimates | PY | PY | No | PY | No | No | No | No | No |
| Overall confidence (modified) | High | High | Moderate | Moderate | Moderate | High | Moderate | Moderate | Moderate |

## Appendix 3: Quality of included systematic reviews based on modified AMSTAR-2 (continued)

| AMSTAR-2 criteria | Schwingshackl 2017b | De Souza 2015 | Pham 2014 | Hooper 2020 | Uusitupa 2019 | Zeraatkar 2019b | Kazemi 2020 | Schwingshackl 2014 |
| --- | --- | --- | --- | --- | --- | --- | --- | --- |
| PICO | Yes | Yes | Yes | Yes | Yes | Yes | Yes | Yes |
| A priori methods | Yes | PY | No | Yes | No | Yes | Yes | No |
| Inclusion | No | Yes | Yes | Yes | Yes | Yes | Yes | No |
| Search strategy | Yes | Yes | Yes | Yes | PY | Yes | Yes | PY |
| Study selection | No | No | No | Yes | No | Yes | No | Yes |
| Data extraction | Yes | Yes | No | Yes | Yes | Yes | Yes | No |
| Study exclusion | PY | PY | No | Yes | PY | PY | Yes | PY |
| Study characteristics | Yes | Yes | Yes | Yes | Yes | Yes | Yes | PY |
| Risk of bias | Yes | Yes | No | Yes | Yes | Yes | Yes | Yes |
| Sources of funding | PY | Yes | No | Yes | No | Yes | PY | No |
| Meta-analysis methods | Yes | Yes | Yes | Yes | Yes | Yes | Yes | Yes |
| Impact of risk of bias | Yes | Yes | No | Yes | Yes | Yes | Yes | Yes |
| Account for risk of bias | Yes | Yes | No | Yes | Yes | Yes | Yes | Yes |
| Heterogeneity and a-priori subgroup | Yes | No | No | Yes | Yes | Yes | No | No |
| Publication bias | Yes | Yes | Yes | Yes | Yes | Yes | Yes | Yes |
| Conflict of interest | Yes | Yes | Yes | Yes | Yes | Yes | Yes | Yes |
| Certainty of evidence | Yes | Yes | No | Yes | Yes | Yes | Yes | Yes |
| Absolute effect estimates | No | PY | No | PY | No | PY | No | No |
| Overall confidence (modified) | Low | Moderate | Critically low | High | Critically low | High | Moderate | Critically low |

PY = Partial yes; PICO = Participants, intervention(s)/exposure(s), comparator, outcome(s)

## Appendix 4: Summary of findings of systematic reviews of observational studies and randomized controlled trials

| **Study** | **Review  quality** | **Study Type**  **(sample size)**  **Follow-up** | **Exposure(s)/ intervention(s)** | **ARR* (per 1000 [95% CI]) of lower versus higher intake** | **CoE** | **Plain Language**** |
| --- | --- | --- | --- | --- | --- | --- |
| **All-cause mortality** | | | | | |  |
| Hooper 2020 | High | RCT | Dietary fat (reduced or replaced) | Very small (5 fewer [11 fewer to 3 more]), p>0.05 | Moderate | Probably leads to little or no difference |
| Zeraatkar 2019a | High | Cohort | Processed meat | Very small (9 fewer [15 to 5 fewer]), p<0.05 | Low | May lead to little or no difference |
| Zeraatkar 2019a | High | Cohort | Red meat | Very small (8 fewer [15 to 0 fewer]), p=0.05 | Very low | It is uncertain whether it leads to a difference |
| Zeraatkar 2019b | High | RCT | Red meat | Very small (1 fewer [7 fewer to 3 more]), p>0.05 | Low | May lead to little or no difference |
| Schwingshackl 2017c | Moderate | Cohort | Dairy | Very small (3 fewer), p>0.05 | Moderate | Probably leads to little or no difference |
| Schwingshackl 2017c | Moderate | Cohort | Processed meat | Small but important (20 fewer), p<0.05 | Moderate | Probably leads to a slight difference |
| Schwingshackl 2017c | Moderate | Cohort | Red meat | Very small (10 fewer), p=0.05 | Moderate | Probably leads to little or no difference |
| De Souza 2015 | Moderate | Cohort | Dietary fat (reduced) | Very small (1 more), p>0.05 | Very low | It is uncertain whether it leads to a difference |
| Schwingshackl 2014 | Critically low | RCT | Dietary fat (replaced fat) | Very small (9 fewer [36 fewer to 28 more]), p>0.05 | Moderate | Probably leads to little or no difference |
| Schwingshackl 2014 | Critically low | RCT | Dietary fat (reduced fat) | Small but important (24 fewer [66 fewer to 54 more]), p>0.05 | Moderate | Probably leads to a slight difference |
| **Cancer mortality (all cancers)** | | | | | | |
| Hooper 2020 | High | RCT | Dietary fat (reduced or replaced) | Very small (0 event [41 fewer to 67 more]), p>0.05 | NR | N/A |
| Zeraatkar 2019b | High | RCT | Red meat | Small but important (12 fewer [26 fewer to 2 more]), p>0.05 | Very low | The evidence is very uncertain |
| Han 2019 | High | Cohort | Red meat | Very small (7 fewer [9 to 6 fewer]), p<0.05 | Low | May lead to little or no difference |
| Han 2019 | High | Cohort | Processed meat | Very small (8 fewer [12 fewer to 6 fewer]), p<0.05 | Low | May lead to little or no difference |
| **Cancer mortality (specific cancers)** | | | | | | |
| Han 2019 (Gastric) | High | Cohort | Processed meat | Very small (1 fewer [1 fewer to 0 more]), p=0.05 | Low | May lead to little or no difference |
| Han 2019 (Colorectal) | High | Cohort | Processed meat | Very small (8 fewer [12 to 6 fewer]), p<0.05 | Very low | The evidence is very uncertain |
| Han 2019 (Pancreatic) | High | Cohort | Processed meat | Very small (1 fewer [3 fewer to 2 more]), p>0.05 | Very low | The evidence is very uncertain |
| Han 2019 (Prostate) | High | Cohort | Processed meat | Very small (1 fewer [2 to 1 fewer]), p<0.05 | Low | May lead to little or no difference |
| Han 2019 (Prostate) | High | Cohort | Red meat | Very small (3 more [0 fewer to 10 more]), p=0.05 | Very low | The evidence is very uncertain |
| Zeraatkar 2019b (breast) | High | RCT | Red meat | Very small (5 fewer [11 fewer to 10 more]), p>0.05 | Very low | The evidence is very uncertain |
| **Cancer incidence (all cancers)** | | | | | | |
| Hooper 2020 | High | RCT | Dietary fat (reduced or replaced) | Very small (11 fewer (31 fewer to 13 more]), p>0.05 | NR | N/A |
| Han 2019 | High | Cohort | Processed meat | Very small [2 fewer (20 fewer to 17 more)], p>0.05 | Very low | The evidence is very uncertain |
| Han 2019 | High | Cohort | Red meat | Very small [13 fewer (31 fewer to 7 more)], p>0.05 | Very low | The evidence is very uncertain |
| **Cancer incidence** **(specific cancers)** | | | | | | |
| Han 2019 (Small intestinal) | High | Cohort | Processed meat | No effect (0 events) | Low | May lead to little or no difference |
| Han 2019 (Pancreatic) | High | Cohort | Processed meat | No effect (0 events) | Low | May lead to little or no difference |
| Han 2019 (Pancreatic) | High | Cohort | Unprocessed red meat | No effect (0 events) | Low | May lead to little or no difference |
| Han 2019 (Oral) | High | Cohort | Processed meat | Very small (1 fewer [2 fewer to 0 more]), p=0.05 | Very low | The evidence is very uncertain |
| Han 2019 (Endometrial) | High | Cohort | Processed meat | Very small (1 fewer [2 fewer to 1 more]), p>0.05 | Very low | The evidence is very uncertain |
| Han 2019 (Ovarian) | High | Cohort | Processed meat | Very small (1 fewer [3 fewer to 1 more]), p>0.05 | Low | May lead to little or no difference |
| Han 2019 (Hepatic) | High | Cohort | Processed meat | Very small (1 more [1 fewer to 4 more]), p>0.05 | Very low | The evidence is very uncertain |
| Han 2019 (Esophageal) | High | Cohort | Processed meat | Very small (2 fewer [3 fewer to 1 fewer]), p<0.05 | Very low | The evidence is very uncertain |
| Han 2019 (Esophageal) | High | Cohort | Red meat | Very small (0 event [2 fewer to 3 more]), p>0.05 | Very low | The evidence is very uncertain |
| Han 2019 (Gastric) | High | Cohort | Processed meat | Very small (2 fewer [5 fewer to 4 more]), p>0.05 | Very low | The evidence is very uncertain |
| Han 2019 (Gastric) | High | Cohort | Red meat | Very small (2 fewer [5 fewer to 3 more]), p>0.05 | Very low | The evidence is very uncertain |
| Han 2019 (Breast) | High | Cohort | Processed meat | Very small (5 fewer [7 fewer to 2 fewer]), p<0.05 | Low | May lead to little or no difference |
| Han 2019 (Breast) | High | Cohort | Red meat | Very small (6 fewer [13 fewer to 3 more]), p>0.05 | Low | May lead to little or no difference |
| Han 2019 (Prostate) | High | Cohort | Processed meat | Very small (0 event [1 fewer to 0 fewer]), p=0.05 | Low | May lead to little or no difference |
| Han 2019 (Prostate) | High | Cohort | Red meat | Very small (1 more [2 fewer to 4 more]), p>0.05 | Low | May lead to little or no difference |
| Han 2019 (Colorectal) | High | Cohort | Processed meat | Very small (1 fewer [2 fewer to 1 fewer]), p<0.05 | Low | May lead to little or no difference |
| Han 2019 (Colorectal) | High | Cohort | Red meat | Very small (0 event [2 fewer to 2 more]), p>0.05 | Low | May lead to little or no difference |
| Kazemi 2021 (Breast) | Moderate | Cohort | Processed meat | Very small (7 fewer), p<0.05 | Moderate | Probably leads to little or no difference |
| Kazemi 2021 (Breast) | Moderate | Cohort | Red meat | Very small (4 fewer), p<0.05 | Moderate | Probably leads to little or no difference |
| Kazemi 2021 (Breast) | Moderate | Cohort | Cheese | Very small (2 more), p=0.05 | Moderate | Probably leads to little or no difference |
| Kazemi 2021 (Breast) | Moderate | Cohort | Dairy | Very small (1 more), p=0.05 | Moderate | Probably leads to little or no difference |
| Schwingshackl 2018 (Colorectal) | Moderate | Cohort | Dairy | Very small (4 more), p<0.05 | Moderate | Probably leads to little or no difference |
| Schwingshackl 2018 (Colorectal) | Moderate | Cohort | Processed meat | Very small (2 fewer), p<0.05 | Moderate | Probably leads to little or no difference |
| Schwingshackl 2018 (Colorectal) | Moderate | Cohort | Red meat | Very small (2 fewer), p<0.05 | Moderate | Probably leads to little or no difference |
| Pham 2014 (Colorectal) | Critically low | Cohort | Processed meat | Very small (3 fewer), p=0.05 | NR | N/A |
| Pham 2014 (Colorectal) | Critically low | Cohort | Red meat | Very small (3 fewer), p>0.05 | NR | N/A |
| Pham 2014 (Colorectal) | Critically low | Cohort | Total meat including poultry | Very small (1 fewer), p>0.05 | NR | N/A |
| **Cardiovascular mortality** | | | | | | |
| Hooper 2020 | High | RCT | Dietary fat (reduced or replaced) | Very small (2 fewer [9 fewer to 5 more]), p>0.05 | Moderate | Probably leads to little or no difference |
| Zeraatkar 2019a | High | Cohort | Processed meat | Very small (4 fewer [7 to 1 fewer]), p<0.05 | Very low | The evidence is very uncertain |
| Zeraatkar 2019a | High | Cohort | Red meat | Very small (4 fewer [5 to 4 fewer]), p<0.05 | Very low | The evidence is very uncertain |
| Zeraatkar 2019b | High | RCT | Red meat | Very small (3 fewer [11 fewer to 8 more]), p>0.05 | Very low | The evidence is very uncertain |
| Schwab 2021 | Moderate | Cohort | Dietary fat (reduced) in T2D population | Small but important (15 more [23 fewer to 67 more]), p>0.05 | Very low | The evidence is very uncertain |
| Schwab 2021 | Moderate | Cohort | Dietary fat (replaced) in T2D population | Small but important (15 fewer [26 to 1 fewer]), p<0.05 | Very low | The evidence is very uncertain |
| Schwab 2021 | Moderate | Cohort | Dietary fat (replaced) in T2D population | Very small (1 fewer [14 fewer to 12 more]), p>0.05 | Very low | The evidence is very uncertain |
| Schwab 2021 | Moderate | Cohort | Dietary fat (replaced) in T2D population | Small but important (20 fewer [37 to 0 fewer]), p=0.05 | Very low | The evidence is very uncertain |
| De Souza 2015 | Moderate | Cohort | Dietary fat (reduced) | Very small (1 more), p>0.05 | Very low | The evidence is very uncertain |
| Schwingshackl 2014 | Critically low | RCT | Dietary fat (replaced) | Very small (2 fewer [14 fewer to 17 more]), p>0.05 | Moderate | Probably leads to little or no difference |
| Schwingshackl 2014 | Critically low | RCT | Dietary fat (reduced) | Very small (3 fewer [14 fewer to 13 more]), p>0.05 | Moderate | Probably leads to little or no difference |
| **Coronary heart disease** | | | | | | |
| Jakobsen 2021 (Fatal and non-fatal) | High | Cohort | Butter | No effect (0 events) | Low | May lead to little or no difference |
| Jakobsen 2021 (Fatal and non-fatal) | High | Cohort | High fat cheese | Very small (4 fewer), p=0.05 | Moderate | Probably leads to little or no difference |
| Hooper 2020 (Fatal) | High | RCT | Dietary fat (reduced or replaced) | Very small (1 fewer [4 fewer to 3 more]), p>0.05 | Low | May lead to little or no difference |
| Hooper 2020 (Fatal and non-fatal) | High | RCT | Dietary fat (reduced or replaced) | Very small (8 fewer [14 fewer to 0 more]), p=0.05 | Very low | The evidence is very uncertain |
| Bechthold 2019 (Fatal and non-fatal) | Moderate | Cohort | Dairy | No effect (0 events) | Moderate | Likely results in little or no difference |
| Bechthold 2019 (Fatal and non-fatal) | Moderate | Cohort | Red meat | Very small (6 fewer), p=0.05 | Moderate | Likely results in little or no difference |
| Bechthold 2019 (Fatal and non-fatal) | Moderate | Cohort | Processed meat | Very small (6 fewer), p<0.05 | Moderate | Likely results in little or no difference |
| De Souza 2015 (Fatal) | Moderate | Cohort | Dietary fat (reduced) | Very small (3 fewer), p>0.05 | Very low | The evidence is very uncertain |
| De Souza 2015 (Fatal and non-fatal) | Moderate | Cohort | Dietary fat (reduced) | Very small (3 fewer), p>0.05 | Very low | The evidence is very uncertain |
| **Combined CVD** | | | | | | |
| Hooper 2020 | High | RCT | Dietary fat (reduced or replaced) | Small but important (16 fewer [26 to 5 fewer]), p<0.05 | Moderate | Probably leads to a slight difference |
| **Stroke** | | | | | | |
| Jakobsen 2021 (Fatal and non-fatal) | High | Cohort | Butter | No effect (0 events) | Low | May lead to little or no difference |
| Jakobsen 2021 (Fatal and non-fatal) | High | Cohort | Cheese | Very small (2 more), p=0.05 | Moderate | Probably leads to little or no difference |
| Hooper 2020 (Fatal and non-fatal) | High | RCT | Dietary fat (reduced or replaced) | Very small (2 fewer [6 fewer to 5 more]), p>0.05 | Very low | The evidence is very uncertain |
| Zeraatkar 2019a (Fatal) | High | Cohort | Processed meat | No effect (0 events) | Very low | The evidence is very uncertain |
| Zeraatkar 2019a (Fatal) | High | Cohort | Red meat | No effect (0 events) | Very low | The evidence is very uncertain |
| Zeraatkar 2019b (Fatal) | High | RCT | Red meat | No effect (0 events) | Very low | The evidence is very uncertain |
| Zeraatkar 2019a (Fatal and non-fatal) | High | Cohort | Processed meat | Very small (1 fewer event [2 to 0 fewer]), p=0.05 | Low | May lead to little or no difference |
| Zeraatkar 2019a (Fatal and non-fatal) | High | Cohort | Red meat | Very small (1 fewer event [2 to 0 fewer]), p=0.05 | Low | May lead to little or no difference |
| Bechthold 2019 (Fatal and non-fatal) | Moderate | Cohort | Red meat | Very small (3 fewer), p<0.05 | Moderate | Probably leads to little or no difference |
| Bechthold 2019 (Fatal and non-fatal) | Moderate | Cohort | Processed meat | Very small (3 fewer), p=0.05 | Moderate | Probably leads to little or no difference |
| Bechthold 2019 (Fatal and non-fatal) | Moderate | Cohort | Dairy | Very small (1 more), p=0.05 | Moderate | Probably leads to little or no difference |
| De Souza 2015 (Fatal and non-fatal) | Moderate | Cohort | Dietary fat (reduced) | 0 events, p>0.05 | Very low | The evidence is very uncertain |
| **Myocardial infarction** | | | | | | |
| Hooper 2020 (Non-fatal) | High | 8 RCTs  (52,834)  55 month follow-up | Dietary fat (reduced or replaced) | Very small (1 fewer [3 fewer to 2 more]), p>0.05 | Low | May lead to little or no difference |
| Hooper 2020 (Fatal and non-fatal) | High | 11 RCTs  (53,167)  55 month follow-up | Dietary fat (reduced or replaced) | Very small (4 fewer [7 fewer to 0 more]), p=0.05 | Very low | The evidence is very uncertain |
| Zeraatkar 2019a (Fatal and non-fatal) | High | Cohort  (55,171)  13.6 years follow-up | Processed meat | Very small (2 fewer [3 fewer to 2 fewer]), p<0.05 | Very low | The evidence is very uncertain |
| Zeraatkar 2019a (Fatal and non-fatal) | High | Cohorts  (55,171)  13.6 years follow-up | Red meat | Very small (3 fewer [5 to 0 fewer]), p=0.05 | Very low | The evidence is very uncertain |
| Schwingshackl 2014 | Critically low | RCT | SFA (reduced or replaced) | Very small (9 fewer [17 fewer to 3 more]), p>0.05 | Moderate | Probably leads to little or no difference |
| Schwingshackl 2014 | Critically low | RCT | SFA (reduced or replaced) | Very small (6 more [4 fewer to 21 more]), p>0.05 | Moderate | Probably leads to little or no difference |
| **Type 2 diabetes** | | | | | | |
| Hooper 2020 | High | RCT | SFA (reduced or replaced) | Very small (2 fewer [6 fewer to 1 more]), p>0.05 | N/R | N/A |
| Zeraatkar 2019a | High | Cohort | Processed meat | Very small (12 fewer [16 fewer to 9 fewer]), p<0.05 | Very low | The evidence is very uncertain |
| Zeraatkar 2019a | High | Cohort | Red meat | Very small (6 fewer [7 to 4 fewer]), p<0.05 | Low | May lead to little or no difference |
| Neuenschwander 2020 | Moderate | Cohort | Dietary fat (reduced) | Very small (2 more), p>0.05 | Low | May lead to little or no difference |
| Schwingshackl 2017a | Moderate | Cohort | Dairy | Very small (5 more), p<0.05 | Moderate | Probably leads to little or no difference |
| Schwingshackl 2017a | Moderate | Cohort | Processed meat | Very small (12 fewer), p<0.05 | High | Leads to little or no difference |
| Schwingshackl 2017a | Moderate | Cohort | Red meat | Very small (10 fewer), p<0.05 | High | Leads to little or no difference |
| De Souza 2015 | Moderate | Cohort | Dietary fat (reduced) | Very small (3 more), p>0.05 | Very low | The evidence is very uncertain |
| Uusitupa 2019 | Critically low | RCT | Dietary fat  (reduced) | Small but important (26 fewer [33 to 18 fewer]), p<0.05 | High | Leads to a slight difference |
| **Health-related quality of life** | | | | | | |
| Hooper 2020 | High | RCT | Dietary fat (reduced or replaced) | Very small, p<0.05 | NR | NA |

CoE = Certainty of evidence

*ARR = Absolute Risk Reduction. We calculated ARR as follows: ARR = baseline risk - corresponding risk. Corresponding risk = effect size*baseline risk. We used data from the Emerging Risk Factors Collaboration (39) and GLOBOCAN (38) to calculate the baseline risks for cardiometabolic and cancer outcomes respectively.

**When the primary meta-analysis compares higher versus lower SF intake, in order to uniformly present findings of lower versus higher intake across studies, we used the reciprocal of the relative effect to estimate absolute effects. Confidence intervals are not presented in such cases, instead we present p values. P values are reported as =0.05 if the upper or lower bound was 0.

***We followed the Cochrane Handbook guidance on effect size language and plain language summaries

**** Grey highlights indicate high quality systematic reviews based on modified AMSTAR-2 criteria

## Appendix 5: Summary of findings of surrogate outcomes

| **References** | **Intervention(s)** | **Comparator(s)** | **Outcomes** | **# of studies (participants)** | **Effect size (95% CI)** | **Certainty of Evidence** |
| --- | --- | --- | --- | --- | --- | --- |
| Hooper 2020 | Dietary fat (reduction or replacement) | Higher intake of SFA (dietary fat) | TG | 7 (3,845) | MD -0.08 mmol/L (-0.21 to 0.04) | NR |
|  |  |  | LDL | 5 (3,291) | MD -0.19 mmol/L (-0.33 to -0.05) | NR |
|  |  |  | HDL | 6 (5,147) | MD -0.01 mmol/L (-0.02 to 0.01) | NR |
|  |  |  | TC | 14 (7,115) | MD -0.24 mmol/L (-0.36 to -0.13) | NR |
|  |  |  | SBP | 5 (3,812) | MD -0.19 mmHg (-1.36 to 0.97) | NR |
|  |  |  | DBP | 5 (3,812) | MD -0.36 mmHg (-1.03 to 0.32) | NR |
|  |  |  | BMI | 6 (43,894) | MD -0.42 [-0.72, -0.12] | NR |

CI = Confidence interval, DBP = Diastolic blood pressure, HDL = High density lipoproteins, LDL = Low density lipoproteins, NR = Not reported, SBP = Systolic blood pressure, SFA = Saturated fatty acid, TC = Total cholesterol

## Appendix 6: Subgroup analysis - all-cause mortality (Hooper et al. 2020)

| **Subgroup by any substitution** | **RR (95% CI)** | **p-value** |
| --- | --- | --- |
| replaced by PUFA | 0.96 [0.82, 1.13] | P = 0.80 |
| replaced by MUFA | 3.00 [0.33, 26.99] |  |
| replaced by CHO | 0.97 [0.90, 1.04] |  |
| replaced by protein | 0.97 [0.90, 1.04] |  |
| replacement unclear | Not estimable |  |
| **Subgroup by duration** | 0.96 [0.90, 1.03] |  |
| up to 24mo | 0.99 [0.78, 1.26] | P = 0.61 |
| >24 to 48mo | 0.96 [0.83, 1.12] |  |
| >48mo | 0.96 [0.79, 1.16] |  |
| unclear duration | 0.33 [0.07, 1.61] |  |
| **Subgroup by baseline SFA** | 0.96 [0.90, 1.03] |  |
| up to 12%E SFA baseline | 0.90 [0.67, 1.21] | P = 0.52 |
| >12 to 15%E SFA baseline | 1.01 [0.86, 1.19] |  |
| >15 to 18%E SFA baseline | 0.35 [0.04, 3.12] |  |
| >18%E SFA baseline | 0.98 [0.83, 1.15] |  |
| unclear | 0.80 [0.62, 1.04] |  |
| **Subgroup by SFA change** | 0.96 [0.90, 1.03] |  |
| up to 4%E difference | 0.99 [0.86, 1.13] | P = 0.37 |
| >4 to 8%E difference | 0.41 [0.08, 2.07] |  |
| >8%E difference | 0.98 [0.83, 1.15] |  |
| unclear | 0.80 [0.62, 1.04] |  |
| **Subgroup by sex** | 0.96 [0.90, 1.03] |  |
| Men | 0.96 [0.83, 1.11] | P = 0.41 |
| Women | 0.97 [0.90, 1.05] |  |
| Mixed, men and women | 0.33 [0.07, 1.61] |  |
| **Subgroup by CVD risk** | 0.96 [0.90, 1.03] |  |
| Low CVD risk | 0.97 [0.91, 1.04] | P = 0.41 |
| Moderate CVD risk | 0.33 [0.07, 1.61] |  |
| Existing CVD disease | 0.97 [0.76, 1.24] |  |
| **Subgroup by TC reduction** | Subtotals only |  |
| serum cholesterol reduced by at least 0.2mmol/L | 0.96 [0.81, 1.14] | P = 0.87 |
| serum cholesterol reduced by <0.2mmol/L | 0.97 [0.90, 1.04] |  |
| serum cholesterol reduction unclear | 0.51 [0.05, 5.46] |  |
|  |  |  |

## Appendix 7: Subgroup analysis – cardiovascular mortality (Hooper et al. 2020)

| **Subgroup by any substitution** | **RR (95% CI)** | **p-value** |
| --- | --- | --- |
| replaced by PUFA | 0.95 [0.73, 1.25] | P = 0.79 |
| replaced by MUFA | 3.00 [0.33, 26.99] |  |
| replaced by CHO | 0.99 [0.85, 1.14] |  |
| replaced by protein | 0.99 [0.86, 1.14] |  |
| replacement unclear | Not estimable |  |
| **Subgroup by duration** | 0.95 [0.78, 1.16] |  |
| up to 24mo | 1.26 [0.54, 2.94] | P = 0.37 |
| >24 to 48mo | 0.79 [0.57, 1.08] |  |
| >48mo | 1.02 [0.73, 1.43] |  |
| unclear duration | 0.25 [0.03, 2.19] |  |
| **Subgroup by baseline SFA** | 0.95 [0.78, 1.16] |  |
| up to 12%E SFA baseline | Not estimable | P = 0.15 |
| >12 to 15%E SFA baseline | 1.06 [0.84, 1.32] |  |
| >15 to 18%E SFA baseline | 0.35 [0.04, 3.12] |  |
| >18%E SFA baseline | 0.70 [0.51, 0.96] |  |
| unclear | 1.00 [0.61, 1.66] |  |
| **Subgroup by SFA change** | 0.95 [0.78, 1.16] |  |
| up to 4%E difference | 1.07 [0.85, 1.33] | P = 0.09 |
| >4 to 8%E difference | 0.29 [0.05, 1.70] |  |
| >8%E difference | 0.70 [0.51, 0.96] |  |
| unclear | 1.00 [0.61, 1.66] |  |
| **Subgroup by sex** | 0.95 [0.78, 1.16] |  |
| Men | 0.96 [0.73, 1.25] | P = 0.45 |
| Women | 1.00 [0.84, 1.19] |  |
| Mixed, men and women | 0.25 [0.03, 2.19] |  |
| **Subgroup by CVD risk** | 0.96 [0.80, 1.14] |  |
| Low CVD risk | 0.84 [0.60, 1.16] | P = 0.26 |
| Moderate CVD risk | 0.25 [0.03, 2.19] |  |
| Existing CVD disease | 1.04 [0.83, 1.31] |  |
| **Subgroup by TC reduction** | Subtotals only |  |
| serum cholesterol reduced by at least 0.2mmol/L | 0.95 [0.73, 1.25] | P = 0.60 |
| serum cholesterol reduced by <0.2mmol/L | 0.97 [0.47, 2.01] |  |
| serum cholesterol reduction unclear | 0.20 [0.01, 4.15] |  |
|  |  |  |

## Appendix 8: Subgroup analysis - myocardial infarction (Hooper et al. 2020)

| **Subgroup by any substitution** | **RR (95% CI)** | **p-value** |
| --- | --- | --- |
| replaced by PUFA | 0.83 [0.67, 1.02] | P = 0.61 |
| replaced by MUFA | 1.40 [0.51, 3.85] |  |
| replaced by CHO | 0.96 [0.86, 1.06] |  |
| replaced by protein | 0.96 [0.86, 1.07] |  |
| replacement unclear | 2.02 [0.19, 21.94] |  |
| **Subgroup by duration** | 0.90 [0.80, 1.01] |  |
| up to 24mo | 0.95 [0.77, 1.17] | P = 0.78 |
| >24 to 48mo | 0.83 [0.64, 1.06] |  |
| >48mo | 0.81 [0.54, 1.24] |  |
| unclear duration | 0.41 [0.02, 7.73] |  |
| **Subgroup by baseline SFA** | 0.90 [0.80, 1.01] |  |
| up to 12%E SFA baseline | Not estimable | P = 0.50 |
| >12 to 15%E SFA baseline | 0.96 [0.87, 1.07] |  |
| >15 to 18%E SFA baseline | 0.52 [0.05, 5.39] |  |
| >18%E SFA baseline | 0.76 [0.55, 1.05] |  |
| unclear | 0.84 [0.54, 1.30] |  |
| **Subgroup by SFA change** | 0.90 [0.80, 1.01] |  |
| up to 4%E difference | 0.96 [0.87, 1.07] | P = 0.50 |
| >4 to 8%E difference | 0.52 [0.05, 5.39] |  |
| >8%E difference | 0.76 [0.55, 1.05] |  |
| unclear | 0.84 [0.54, 1.30] |  |
| **Subgroup by sex** | 0.90 [0.80, 1.01] |  |
| Men | 0.85 [0.73, 0.98] | P = 0.35 |
| Women | 0.97 [0.86, 1.09] |  |
| Mixed, men and women | 0.75 [0.13, 4.47] |  |
| **Subgroup by CVD risk** | 0.90 [0.80, 1.01] |  |
| Low CVD risk | 0.90 [0.72, 1.13] | P = 0.96 |
| Moderate CVD risk | 0.75 [0.13, 4.47] |  |
| Existing CVD disease | 0.87 [0.74, 1.03] |  |
| **Subgroup by TC reduction** | Subtotals only |  |
| serum cholesterol reduced by at least 0.2mmol/L | 0.83 [0.70, 0.98] | P = 0.12 |
| serum cholesterol reduced by <0.2mmol/L | 0.98 [0.87, 1.10] |  |
| serum cholesterol reduction unclear | Not estimable |  |
|  |  |  |

## Appendix 9: Subgroup analysis - myocardial infarction (non-fatal) (Hooper et al. 2020)

| **Subgroup by any substitution** | **RR (95% CI)** | **p-value** |
| --- | --- | --- |
| replaced by PUFA | 0.80 [0.63, 1.03] | P = 0.81 |
| replaced by MUFA | 1.20 [0.42, 3.45] |  |
| replaced by CHO | 0.93 [0.72, 1.21] |  |
| replaced by protein | 0.93 [0.72, 1.21] |  |
| replacement unclear | 2.02 [0.19, 21.94] |  |
| **Subgroup by duration** | 0.97 [0.87, 1.07] |  |
| up to 24mo | 0.83 [0.57, 1.22] | P = 0.52 |
| >24 to 48mo | 0.82 [0.53, 1.27] |  |
| >48mo | 0.99 [0.88, 1.12] |  |
| unclear duration | Not estimable |  |
| **Subgroup by baseline SFA** | 0.97 [0.87, 1.07] |  |
| up to 12%E SFA baseline | Not estimable | P = 0.42 |
| >12 to 15%E SFA baseline | 0.97 [0.83, 1.13] |  |
| >15 to 18%E SFA baseline | Not estimable |  |
| >18%E SFA baseline | 0.62 [0.31, 1.21] |  |
| unclear | 0.91 [0.65, 1.27] |  |
| **Subgroup by SFA change** | 0.97 [0.87, 1.07] |  |
| up to 4%E difference | 0.97 [0.83, 1.13] | P = 0.42 |
| >4 to 8%E difference | Not estimable |  |
| >8%E difference | 0.62 [0.31, 1.21] |  |
| unclear | 0.91 [0.65, 1.27] |  |
| **Subgroup by sex** | 0.97 [0.87, 1.07] |  |
| Men | 0.81 [0.63, 1.03] | P = 0.24 |
| Women | 1.01 [0.90, 1.13] |  |
| Mixed, men and women | 2.02 [0.19, 21.94] |  |
| **Subgroup by CVD risk** | 0.95 [0.80, 1.13] |  |
| Low CVD risk | 0.87 [0.68, 1.12] | P = 0.61 |
| Moderate CVD risk | 2.02 [0.19, 21.94] |  |
| Existing CVD disease | 1.00 [0.76, 1.31] |  |
| **Subgroup by TC reduction** | Subtotals only |  |
| serum cholesterol reduced by at least 0.2mmol/L | 0.80 [0.62, 1.03] | P = 0.10 |
| serum cholesterol reduced by <0.2mmol/L | 1.01 [0.90, 1.13] |  |
| serum cholesterol reduction unclear | Not estimable |  |
|  |  |  |

## Appendix 10: Subgroup analysis - coronary heart disease (fatal and non-fatal) (Hooper et al. 2020)

| **Subgroup by any substitution** | **RR (95% CI)** | **p-value** |
| --- | --- | --- |
| replaced by PUFA | 0.76 [0.57, 1.00] | P = 0.33 |
| replaced by MUFA | 1.50 [0.62, 3.61] |  |
| replaced by CHO | 0.93 [0.78, 1.11] |  |
| replaced by protein | 0.96 [0.88, 1.05] |  |
| replacement unclear | 2.93 [0.31, 27.84] |  |
| **Subgroup by duration** | Subtotals only |  |
| up to 24mo | 1.01 [0.76, 1.35] | P = 0.70 |
| >24 to 48mo | 0.79 [0.55, 1.13] |  |
| >48mo | 0.85 [0.63, 1.15] |  |
| unclear duration | 0.60 [0.10, 3.58] |  |
| **Subgroup by baseline SFA** | Subtotals only |  |
| up to 12%E SFA baseline | Not estimable | P = 0.12 |
| >12 to 15%E SFA baseline | 0.96 [0.88, 1.06] |  |
| >15 to 18%E SFA baseline | 0.31 [0.10, 1.01] |  |
| >18%E SFA baseline | 0.77 [0.56, 1.04] |  |
| unclear | 0.78 [0.49, 1.26] |  |
| **Subgroup by SFA change** | Subtotals only |  |
| up to 4%E difference | 0.96 [0.88, 1.06] | P = 0.12 |
| >4 to 8%E difference | 0.31 [0.10, 1.01] |  |
| >8%E difference | 0.77 [0.56, 1.04] |  |
| unclear | 0.78 [0.49, 1.26] |  |
| **Subgroup by sex** | Subtotals only |  |
| Men | 0.84 [0.70, 1.02] | P = 0.44 |
| Women | 0.97 [0.87, 1.07] |  |
| Mixed, men and women | 0.88 [0.18, 4.36] |  |
| **Subgroup by CVD risk** | Subtotals only |  |
| Low CVD risk | 0.90 [0.76, 1.05] | P = 0.95 |
| Moderate CVD risk | 0.88 [0.18, 4.36] |  |
| Existing CVD disease | 0.94 [0.75, 1.16] |  |
| **Subgroup by TC reduction** | Subtotals only |  |
| serum cholesterol reduced by at least 0.2mmol/L | 0.76 [0.58, 0.99] | P = 0.08 |
| serum cholesterol reduced by <0.2mmol/L | 0.97 [0.88, 1.08] |  |
| serum cholesterol reduction unclear | Not estimable |  |
|  |  |  |

## Appendix 11: Subgroup analysis - coronary heart disease (fatal) (Hooper et al. 2020)

| **Subgroup by any substitution** | **RR (95% CI)** | **p-value** |
| --- | --- | --- |
| replaced by PUFA | 0.98 [0.74, 1.28] | P = 0.80 |
| replaced by MUFA | 3.00 [0.33, 26.99] |  |
| replaced by CHO | 0.99 [0.85, 1.16] |  |
| replaced by protein | 0.99 [0.85, 1.16] |  |
| replacement unclear | Not estimable |  |
| **Subgroup by duration** | Subtotals only |  |
| up to 24mo | 1.02 [0.78, 1.33] | P = 0.35 |
| >24 to 48mo | 0.87 [0.64, 1.19] |  |
| >48mo | 1.02 [0.72, 1.45] |  |
| unclear duration | 0.09 [0.01, 1.60] |  |
| **Subgroup by baseline SFA** | Subtotals only |  |
| up to 12%E SFA baseline | Not estimable | P = 0.38 |
| >12 to 15%E SFA baseline | 1.07 [0.86, 1.34] |  |
| >15 to 18%E SFA baseline | Not estimable |  |
| >18%E SFA baseline | 0.82 [0.55, 1.21] |  |
| unclear | 0.85 [0.56, 1.29] |  |
| **Subgroup by SFA change** | Subtotals only |  |
| up to 4%E difference | 1.07 [0.86, 1.34] | P = 0.38 |
| >4 to 8%E difference | Not estimable |  |
| >8%E difference | 0.82 [0.55, 1.21] |  |
| unclear | 0.85 [0.56, 1.29] |  |
| **Subgroup by sex** | Subtotals only |  |
| Men | 0.98 [0.79, 1.23] | P = 0.27 |
| Women | 0.99 [0.82, 1.20] |  |
| Mixed, men and women | 0.09 [0.01, 1.60] |  |
| **Subgroup by CVD risk** | Subtotals only |  |
| Low CVD risk | 0.95 [0.78, 1.16] | P = 0.23 |
| Moderate CVD risk | 0.09 [0.01, 1.60] |  |
| Existing CVD disease | 1.03 [0.83, 1.27] |  |
| **Subgroup by TC reduction** | Subtotals only |  |
| serum cholesterol reduced by at least 0.2mmol/L | 0.96 [0.75, 1.24] | P = 0.85 |
| serum cholesterol reduced by <0.2mmol/L | 0.99 [0.82, 1.20] |  |
| serum cholesterol reduction unclear | Not estimable |  |
|  |  |  |

## Appendix 12: Subgroup analysis - stroke (fatal and non-fatal) (Hooper et al. 2020)

| **Subgroup by any substitution** | **RR (95% CI)** | **p-value** |
| --- | --- | --- |
| replaced by PUFA | 0.68 [0.37, 1.27] | P = 0.99 |
| replaced by MUFA | Not estimable |  |
| replaced by CHO | 0.73 [0.29, 1.87] |  |
| replaced by protein | 0.65 [0.15, 2.75] |  |
| replacement unclear | 1.01 [0.06, 15.93] |  |
| **Subgroup by duration** | 0.91 [0.67, 1.23] |  |
| up to 24mo | 1.01 [0.06, 15.93] | P = 0.16 |
| >24 to 48mo | 0.57 [0.30, 1.11] |  |
| >48mo | 1.03 [0.91, 1.16] |  |
| unclear duration | 0.20 [0.02, 1.68] |  |
| **Subgroup by baseline SFA** | 0.91 [0.67, 1.23] |  |
| up to 12%E SFA baseline | Not estimable | P = 0.62 |
| >12 to 15%E SFA baseline | 0.91 [0.50, 1.66] |  |
| >15 to 18%E SFA baseline | 0.35 [0.01, 8.12] |  |
| >18%E SFA baseline | 0.59 [0.30, 1.15] |  |
| unclear | 2.00 [0.18, 21.89 |  |
| **Subgroup by SFA change** | 0.91 [0.67, 1.23] |  |
| up to 4%E difference | 0.91 [0.50, 1.66] | P = 0.62 |
| >4 to 8%E difference | 0.35 [0.01, 8.12] |  |
| >8%E difference | 0.59 [0.30, 1.15] |  |
| unclear | 2.00 [0.18, 21.89] |  |
| **Subgroup by sex** | 0.91 [0.67, 1.23] |  |
| Men | 0.63 [0.33, 1.18] | P = 0.16 |
| Women | 1.03 [0.91, 1.16] |  |
| Mixed, men and women | 0.37 [0.07, 1.97] |  |
| **Subgroup by CVD risk** | 1.00 [0.89, 1.11] |  |
| Low CVD risk | 0.86 [0.52, 1.42] | P = 0.42 |
| Moderate CVD risk | 0.37 [0.07, 1.97] |  |
| Existing CVD disease | 1.01 [0.86, 1.18] |  |
| **Subgroup by TC reduction** | Subtotals only |  |
| serum cholesterol reduced by at least 0.2mmol/L | 0.70 [0.38, 1.28] | P = 0.93 |
| serum cholesterol reduced by <0.2mmol/L | 0.65 [0.15, 2.75] |  |
| serum cholesterol reduction unclear | Not estimable |  |
|  |  |  |

## Appendix 13: Subgroup analysis - combined cardiovascular events (Hooper et al. 2020)

| **Subgroup by any substitution** | **RR (95% CI)** | **p-value** |
| --- | --- | --- |
| replaced by PUFA | 0.73 [0.58, 0.92] | P = 0.13 |
| replaced by MUFA | 1.00 [0.53, 1.89] |  |
| replaced by CHO | 0.84 [0.67, 1.06] |  |
| replaced by protein | 0.97 [0.91, 1.03] |  |
| replacement unclear | 1.68 [0.41, 6.87] |  |
| **Subgroup by duration** | 0.79 [0.66, 0.93] |  |
| up to 24mo | 0.96 [0.78, 1.16] | P = 0.19 |
| >24 to 48mo | 0.73 [0.56, 0.95] |  |
| >48mo | 0.85 [0.63, 1.16] |  |
| unclear duration | 0.43 [0.17, 1.08] |  |
| **Subgroup by baseline SFA** | 0.79 [0.66, 0.93] |  |
| up to 12%E SFA baseline | Not estimable | P = 0.008 |
| >12 to 15%E SFA baseline | 0.97 [0.91, 1.03] |  |
| >15 to 18%E SFA baseline | 0.41 [0.22, 0.78] |  |
| >18%E SFA baseline | 0.79 [0.63, 1.00] |  |
| unclear | 0.72 [0.51, 1.03] |  |
| **Subgroup by SFA change** | 0.79 [0.66, 0.93] |  |
| up to 4%E difference | 0.97 [0.91, 1.03] | P = 0.005 |
| >4 to 8%E difference | 0.40 [0.22, 0.74] |  |
| >8%E difference | 0.79 [0.63, 1.00] |  |
| unclear | 0.72 [0.51, 1.03] |  |
| **Subgroup by sex** | 0.79 [0.66, 0.93] |  |
| Men | 0.80 [0.69, 0.93] | P = 0.03 |
| Women | 0.98 [0.92, 1.04] |  |
| Mixed, men and women | 0.59 [0.23, 1.49] |  |
| **Subgroup by CVD risk** | 0.83 [0.72, 0.96] |  |
| Low CVD risk | 0.89 [0.75, 1.06] | P = 0.67 |
| Moderate CVD risk | 0.59 [0.23, 1.49] |  |
| Existing CVD disease | 0.86 [0.71, 1.05] |  |
| **Subgroup by TC reduction** | Subtotals only |  |
| serum cholesterol reduced by at least 0.2mmol/L | 0.74 [0.59, 0.92] | p=0.03 |
| serum cholesterol reduced by <0.2mmol/L | 0.98 [0.91, 1.04] |  |
| serum cholesterol reduction unclear | 0.20 [0.01, 4.15] |  |
|  |  |  |
